# Genomes 2000 International Conference on Microbial and Model Genomes

**DOI:** 10.1002/1097-0061(20000630)17:2<124::AID-YEA23>3.0.CO;2-L

**Published:** 2000

**Authors:** Jo Wixon

**Affiliations:** School of Biological Sciences University of Manchester Manchester M13 9PT UK


**Yeast**
*Yeast* 2000; **17**: 124–133.

**Meeting Highlights**

### Genomes 2000 International Conference on Microbial and Model Genomes http://www.pasteur.fr/recherche/unites/gmp
http://www.asmusa.org

**The meeting was held on 11–15 April at the Institut Pasteur in Paris and was co-organized by the American Society for Microbiology (ASM) and Institut Pasteur.**

Jo Wixon
*Managing Editor—School of Biological Sciences, University of Manchester, Manchester, M13 9PT, UK*

### Summary

The Genomes 2000 meeting covered a broad range of organisms, with the emphasis being placed on microbes, in line with the interests of the Institut Pasteur and the ASM. The talks in the first session gave a taster of some of the eagerly awaited eukaryote genomes, several of which are nearing completion. In the second session, a selection of bacterial genomes were described and contrasted with each other, giving interesting insights into microbial physiology and evolution. The later sessions focused on the approaches being applied to whole genomes. There were representatives from databases who described how their resources are moving with the times, those developing or improving functional genomics tools or running large-scale functional genomics projects reported back on progress and described future plans, researchers using structural genomics demonstrated the power of this technique both in the lab and at the computer, and those working on evolution and comparative genomics gave us food for thought on many topics related to phylogeny and mechanisms of genome evolution. The meeting may have started with the 'Clash of the Titans' but it went on to show how smaller projects, in many cases undertaken by networks of laboratories, can also achieve important results. There are genomes of all shapes and sizes out there waiting to be sequenced and many genes whose functions we have yet to determine. As we start the new millenium, this is a rapidly growing field and there are many exciting challenges to face.

Stewart Cole opened the meeting with a welcome address given on behalf of the Director General of the Institut Pasteur, in which he described the history of the Institut Pasteur. The first genome sequence to be released by the Institut was that of human papillomavirus. Since then they have completed the genomes of HIV1 and 2 and contributed to the sequencing of *Saccharomyces cerevisiae*, *Bacillus subtilis* and *Mycobacterium tuberculosis*. This was followed by a welcome from the ASM given by Noel Rose (Johns Hopkins University, USA), who once worked at Institut Pasteur. The ASM sponsored the attendance of several students and young post-docs.

### Session A — Human and eukaryotic genome sequencing

The first speaker, Craig Venter (Celera Genomics, USA) gave an overview of the contibutions of The Institute for Genomic Research (TIGR) and Celera to the sequencing community, highlighting their work on ***Drosophila*** and ***human***. Celera recently completed the *Drosophila* genome, which took 2.4 million reads. They then held an 'Annotation Jamboree', in which a large team of *Drosophila* specialists and bioinformaticians assessed the results of the gene prediction programs and attempted to functionally categorize each predicted gene. 48% of the genes had database matches but were of unknown function and 11% were unique (hypotheticals); these are the potential 'fly-specific' genes. They plan to try to obtain expression data or EST sequences representing these genes. Celera's human genome sequence is now in the assembly and gap-

Copyright © 2000 John Wiley & Sons, Ltd.

closure phase for one male individual, and the sequencing of a further five individuals (an ethnically diverse set including four females) is under way. Any one human is heterozygous for ~3 million SNPs; they hope to gain information on ~6million SNPs from the six sequences. Although he did say that the human genome sequence would be released after publication of the paper, Venter made no comment as to the format of the sequence or what conditions might be placed on access. Celera have also 'officially' started the mouse genome. They feel that comparing this to human will be a powerful tool for discovering regulatory regions, a task not yet tackled with any great success by search algorithms. There are also plans for a proteomics project. This will involve production of antibodies to all proteins, as tools to determine protein–protein interactions, and using mass spectrometry to identify proteins separated from complex mixtures.

Jean Weissenbach (Genoscope, France) talked about the past, present and future of the **public human genome project**. To date there are already ~2600 Mb of human sequence in the public databases, which represents 80% of the human genome. The working draft which they intend to release in May 2000 will have 5X random shotgun coverage of the BAC contigs. Version 2, which is planned for 2001, will have ordered and oriented contigs. They hope to have over 40 000 SNPs on the third-generation genome map. His group are also trying to circumvent the difficulties of human gene prediction using data from *Tetraodon nigroviridis*, a more easily reared relative of *Fugu* (the pufferfish). They have sequenced about one-third of the genome, which allows them to find almost two-thirds of the genes-using their prediction tool, EXOFISH. By comparing this data with the human sequence, they can detect evolutionarily conserved regions, which they call 'ecores'. This has allowed them to detect several ORFs not detected by the programs used to predict ORFs on chromosome 22. Using the number of ecores they find per human gene and the level of coverage they have of the *T. nigroviridis* genome, they estimate that there are only 30 000–35 000 human genes, and Weissenbach says that he is confident that there will turn out to be less than 40 000 human genes. By assessing the number of ecores which match UNIGENE entries, he and his team think that it only gives 48% coverage of human genes.

Stephan Beck updated the audience on the status of the **human genome effort at the Sanger Centre** (UK). The group are working on chromosomes 1, 6, 9, 10, 13, 20 and X and have recently completed and published chromosome 22, using a clone-by-clone approach. The Sanger Centre mainly use ABI3700 capillary sequencers and make their data available within 24 hours of assembly. The target for accuracy is less than one error per 10 000 bases; to monitor this, they assign a certainty score to each base of sequence. These can later be used to identify SNPs, which the Sanger Centre is doing as part of the recently announced SNP consortium. The Sanger Centre are using the MHC as the model for an 'epigenome' project, in which they will look for methylated loci. He pointed out that 5-methylcytosine is a part of our DNA and that we commonly forget that DNA therefore has five functional bases. They also plan to make isochore maps, which will detail the local GC content; replicon maps, which will relate to how the genome replicates; and scaffold maps, which will show how the DNA is folded. Work by Denise Sheer has already shown that chromosome 6 exists as a discrete clump in the interphase nucleus and that the MHC appears to be all on one protruding loop.

An overview of the progress of the ***Arabidopsis thaliana* genome sequencing project** was given by Marcel Salanoubat (Genoscope, France). His group is part of an international consortium of laboratories. They are using a strategy involving end sequencing of BACs. Chromosome 3 is predicted to be 24.2 Mb long; they have completed 22.2 Mb and the remaining 2 Mb is in the finishing process. Based on the data they have so far, the average *Arabidopsis* gene has five exons and four introns, and ~22% of the genes are intronless. 40% of the predicted genes are supported by matches with ESTs. One-tenth of the genes found so far have known function, a further 50% have strong homology to another known gene, and the remaining 40% have no clear functional assignment.

TIGR have recently published the completed sequence of ***Arabidopsis thaliana* chromosome 2**. Samir Kaul (TIGR, USA) discussed the expectations researchers have from the completion of the genome. TIGR maintain gene indices for several organisms, which are made by building ESTs into 'unigene-like' contigs. By comparing the gene indices against each other, they are constructing an

Copyright © 2000 John Wiley & Sons, Ltd. 

orthologue database. They are also producing an annotation database, in which they aim to categorize genes by their probable function. This will be searchable by BLAST and with keyword queries. These databases aid in gene prediction by providing evidence of expression. TIGR are also starting a microarray project for the ORFs predicted from chromosome 2, which should provide expression data to verify some of the hypothetical ORFs.

**Rice** (*Oryza sativa*) is an important crop, which plays a crucial role in several economies. Takuji Sasaki (NIAR, Japan) gave a presentation on the **sequencing** of this 430 Mb genome by an international consortium of labs, including the Japanese Rice Genome Project (RGP). The consortium now has 30–40% coverage using PAC clones. ~5000 ESTs have been placed on the map, and a further 2000 are available as probes to identify clones to fill the gaps. The ESTs will be used to help with the annotation, in combination with BLAST searches of the existing databases and LTR searches. The exon boundary prediction will use *Arabidopsis* and maize data to detect splice sites, since very little relevant data exists for rice. RGP are constructing a new database, 'INE' (Japanese for 'rice plant'), to handle the 30 000–40 000 genes they expect to find in the rice genome. Rice genes appear to be smaller than expected, the average protein so far being 200 amino acids long. Last week, Monsanto offered to assist in the sequencing of the genome. This is seen as an exciting opportunity to speed up the project. Future plans include a gene disruption project using a retrotransposon insertion system.

Researchers at Baylor College, USA, are working on a project to **sequence the genome of *Dictyostelium discoideum*** with the Sanger Centre and the Institute of Molecular Biotechnology at Jena. Adam Kuspa presented the progress that has been made so far. *Dictyostelium* is a soil amoeba that feeds on bacteria. On starvation, it forms a structure in which 50–1000 cells aggregate, and this then forms a ball of spores (a fruiting body). The 34 Mb genome is distributed over six chromosomes, is fairly AT-rich (at ~77%) and encodes ~8000 genes. The GC content in coding regions is ~33%, falling to ~10% in non-coding regions, this has been exploited to aid gene prediction. As much as 10% of the genome is complex repeats, including 100 copies of the rDNA palindrome, many large gene families and tandem duplications. Although there are also many repeat elements, comparisons of those encountered so far indicate that there is adequate variation to allow sequence assembly. Microarrays are already in use to study gene expression patterns, there is an insertional mutagenesis strategy available and mutant cells can be mixed with other cells to produce a mobile 'slug' which searches for food. This can be used to follow the fate of a cell and the lineage of particular cells, in the formation of the fruiting body.

### Session B — Bacterial genome sequencing

Clare Fraser (TIGR) gave an overview of **microbial genome sequencing**. Currently ~100 microbial genomes are complete or are nearing completion. On average, 40–50% of the ORFs predicted from microbial genomes have no known function and, of these, typically 10–30% are unique to the organism of interest. Paralogous ORFs are a common feature of microbe genomes, forming 12–50% of each genome. This effect is most pronounced in the larger genomes, such as that of *E. coli*, and in *Chlamydia pneumoniae*, redundancy is mainly due to tandem repeats. The genome size and GC content of microbial genomes varies greatly, the latter influencing codon usage and amino acid composition. Although sequence comparisons can be used to assess relatedness, gene evolution does not equal species evolution. A major cause for this is the fairly common occurrence of horizontal gene transfer. However, 'average' phylogenetic trees plotted using the whole genome may allow the recovery of the basic phylogenetic history of each species.

Pascale Cossart and Philippe Glaser (Institut Pasteur, France) gave a joint presentation on the results of the analysis of the **genome of *Listeria monocytogenes***. Almost all of the virulence factors have been found within a 10 kb pathogenicity island, which includes genes involved in all of the later stages of infection, whereas the genes involved in entry are located in a different chromosomal region. The sequencing has identified new surface protein genes, such as novel members of the LPXTG family (there are now 40 members), lipotechoic and techoic acid-associated proteins and cell wall and membrane proteins. The group is also sequencing *Listeria innocua*, a non-virulent strain. Some of the LPXTG genes are absent in

Copyright © 2000 John Wiley & Sons, Ltd.

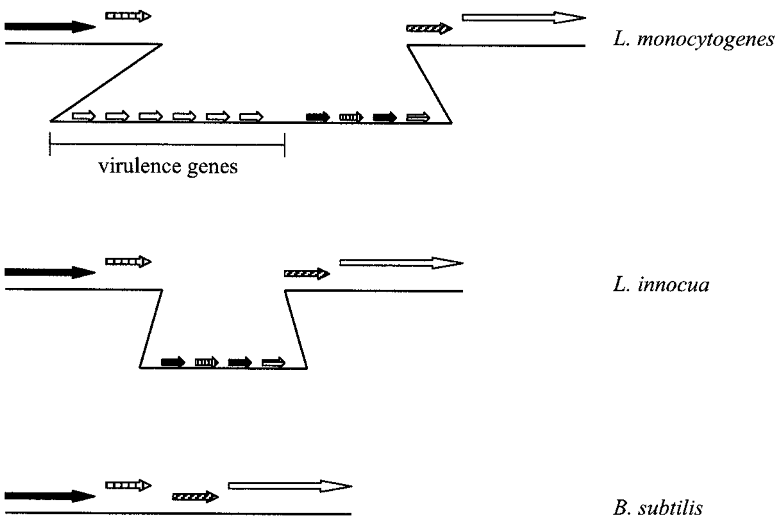

**Figure 1.** A schematic of the comparison of the virulence gene region of *L. monocytogenes* to the syntenic regions of the *L. innocua* and *B. subtilis* genomes (adapted from a presentation by P. Cossart)

the *L. innocua* genome, as are two new techoic-acid-associated proteins from the InlB internalin family. Comparison of the two *Listeria* genomes with that of their close relative *Bacillus subtilis* has shown that a region including the virulence genes may have been inserted into (or lost from) the *B. subtilis* genome (Figure 1). Only some of the 'inserted genes' are present in *L. innocua*, which could indicate two independent insertion events (or losses) occurred to make *L. monocytogenes*, or a single insertion occurred, followed by a subsequent loss of the virulence genes in *L. innocua*, causing it to be avirulent. The comparison with *B. subtilis* is being taken further. For example, in contrast to *B. subtilis, Listeria* cannot sporulate and is not competent, despite having all the genes for this latter function. *B. subtilis* has 4107 genes compared to the 2932 of *L. monocytogenes*, there is a high degree of synteny and 2254 genes are found in both microbes. Both microbes exhibit a strong preference for transcription in the direction of replication.

Jeffrey Miller (UCLA, USA) gave a presentation on the findings of a study into the genome of ***Pyrobaculum aerophilum***. This marine archaean has an optimal growth temperature of 100°C. Analysis of the 2400 ORFs has shown some interesting features of its gene complement and transcription. *Pyrobaculum* does not appear to have a full complement of DNA repair genes and has no recombination repair, mismatch repair or photoreactivation pathways, showing only a functional alkylation reversal mechanism. This seems a risky way to live, and Miller proposes that *Pyrobaculum* must either have higher fidelity enzymes to perform the functions that can cause mismatches or an as-yet undiscovered new family of repair proteins. A particular problem for organisms living at high temperatures is the deamination of cytosine to uracil. By homology searches, the group could not find a *Pyrobaculum* uracil DNA glycosylase. However, by biochemical methods, they isolated the activity and have uncovered a family with a totally different sequence signature, but which folds to give the same secondary structure as the existing family.

***Xylella fastidiosa*** was the first plant pathogen to be sequenced. Andrew Simpson (Ludwig Institute of Cancer Research, Brazil) presented the findings of the project. This pathogen attacks citrus fruits, blocking the xylem and causing production of hard, almost juiceless, fruits of no commercial value. The majority of the ~2.7 Mb genome exists as one large chromosome and there are two small plasmids. 88% of the genome is coding sequence and analysis of the genes has shown that just over half of them have no known function. *Xylella* has a complete complement of TCA cycle and glycolytic enzymes as well as those for amino acid and nucleic acid synthesis. It produces an extracellular polysaccharide which is thought to be important for the aggregation which blocks the xylem. It has several afimbrial adhesins, haemagglutinin-like genes and

Copyright © 2000 John Wiley & Sons, Ltd. 

haemolysin-like genes, which had only been found in animal pathogens prior to their discovery in this, the first plant pathogen to be sequenced. ~5% of the genome is comprised of five extended regions of ds phage homology; these are thought to have been acquired by lateral transfer. The team aim to carry out further comparative analyses, their main aim being to define the genes responsible for pathogenicity. They have also set their eights on *Xanthomonas citri* as their next target for sequencing.

***Streptomyces coelicolor*** is an actinomycete named for the filamentous fungus-like appearance of its colonies and the sky-blue antibiotic they produce. Stephen Bentley (The Sanger Centre, UK) spoke about the sequencing project, which is now nearing completion, with almost all 8 Mb sequenced and work under way to clear the annotation bottleneck. The genome is 90% coding sequence, with ~7400 genes predicted. Most of the genes encoding essential functions are located close to the centromere, with several apparently non-essential genes, rather than 'junk', towards the telomeres. Although *S. coelicolor* has more regulators, membrane transporters, and enzymes than *E. coli*, the ratios of genes in these categories is the same. They think that this could just be *S. coelicolor* needing to have more complex systems to deal with its more complex lifestyle and environment. For example, *S. coelicolor* makes a molecule composed of an 11 amino acid chain that is made into a ring. This is completely catalysed by a complex of proteins, not by ribosomes and the genes responsible take up more than 1% of the genome.

Dusko Ehrlich (INRA, France) gave a presentation on the **genome analysis of lactic acid bacteria**. These bacteria convert carbohydrates into lactic acids via glycolysis and do not use respiration. They are used in milk production and the fermentation of meat vegetables. *Lactococci* form 30% of the strains used in industry, mainly for the production of cheese. *Lactococcus lactis* is a Gram-positive eubacterium that does not make spores and is non-motile. The 2.3 Mb genome is AT-rich (35% GC), with a GC skew and uneven distribution of Chi sites and two types of IS sequences. There are three hotspots of phage integration. Of the 2322 genes, 25% have matches but no known function and 18% have no match at all in the databases; these are presumed to be *Lactococcus*-specific. Comparison to *B. subtilis* shows that many genes are conserved between the two bacteria, with a mean sequence conservation of 40%. The two genomes have the same proportion of regulators (~6%) and counterparts of the competence genes can be found in *Lactococcus*. *Lactococcus* does have some parts of the aerobic respiration pathway; it is able to modify haem, but not to make it. Supplementing cultures with haem does give better growth, possibly by allowing *Lactococcus* to use haem as a co-factor for aerobic respiration.

### Session C — computational genomics

Ross Overbeek (Integrated Genomics, USA) presented a system that uses **clustering of genes on microbial chromosomes to predict function**. This model uses the observation that genes from the same metabolic pathway are often located close together on bacterial chromosomes and then adds in comparative mapping to increase the significance. This requires many sequenced microbial genomes, and uses distantly related species (e.g. eubacteria vs. archaea) to give more significance to a prediction. The program uses genes with a gap of less than 300 bp between them and looks for pairs of bi-directional hits in a wide range of genomes (Figure 2). The more times a pair appears together, the better the score. In identifying such regions containing known genes, they find that in several species, they include genes of unknown function. This allows the prediction that the novel genes are members of the same pathway as the known genes. The model has been proved using the chorismate synthesis pathway, in which the program has discovered a new shikimate kinase and a homoserine kinase. Overbeek calculates that 35–55% of genes occur in close proximity to a gene with a function in the same pathway and that ~10% of hypotheticals can be coupled to genes of assigned function in this way. Currently, he admits that it is harder to spot regulatory genes (which are less conserved in distant species) but he feels that as more closely related species are sequenced, this may well become possible.

Peer Bork described a similar system being used at EMBL to improve the annotation of the databases made available there. The STRING server uses an iterative process to **look for genes with conserved neighbourhood to assign function**. The program takes a starting gene and steps outwards, one gene at a time, looking for matches across many genomes, to

Copyright © 2000 John Wiley & Sons, Ltd.

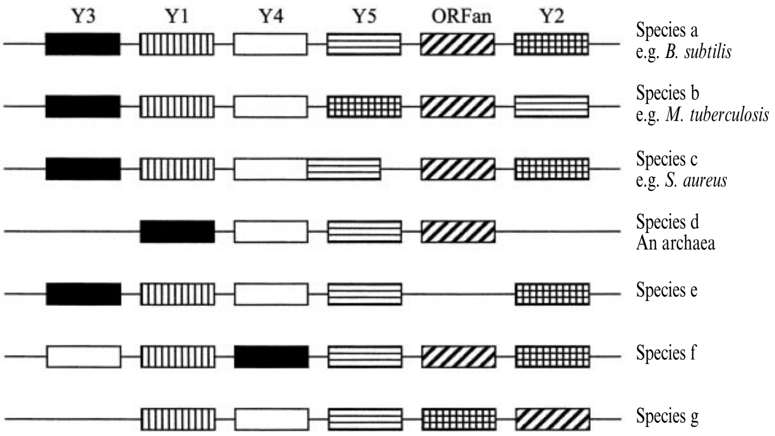

**Figure 2.** A schematic illustrating the use of bi-directional hits to assemble an 'operon', a run of genes (Y1-5) with roles in the same pathway (Y). The diagonally hatched ORFan, is commonly found with genes Y1-5, hence we can infer that this gene has a role in pathway Y. This inference becomes stronger when more distantly related species are used, particularly the archaea, species d build up a picture of the neighbourhood. The scores combine the level of neighbourhood conservation and sequence conservation between species. They are also using data on shared regulation to infer related function. For example, if an ORFan occurs as a gene fusion in one species, this could indicate a linked function for that ORFan which may also be employed in other species.

Julio Collado-Vides (UNAM, Mexico) spoke about a computational approach for **defining all of the regulators and operons of *E. coli***. This approach combines sequence data with literature reports and transcriptome data to identify new operons and regulators. Operons are a subset of all the stretches of genes transcribed in the same direction called 'runs'. Operons have very small intergenic regions. In fact, the termination and initiation codons of genes in operons often overlap, which can be used to detect potential operons within runs. Transcriptome data can then be used to verify operons by looking to see if mRNA levels for all of the genes go up or down together. However, this is not a black and white situation. Even under conditions of strong induction, not every gene in an operon will exhibit the same proportional change in expression. From analysis of the data available, the group predict $\sim 350$ transcriptional regulators, organized into 20 families. Almost half of these have been experimentally characterized; the rest are predicted (mainly due to the presence of a helix–turn–helix motif) and remain to be proven.

### Session D — functional genomics

Minoru Kanehisa (Kyoto University, Japan) presented developments of and plans for the KEGG database to allow it to be used to **predict biochemical pathways using complete genome sequences and microarray data**. The KEGG database has a vast collection of metabolic and regulatory pathway information on a broad range of organisms and is now working with expression profile data. They are looking at new ways of correlating all the data held on each set of clustered genes, such as the application of graph comparisons and path computation algorithms to the reconstruction of biochemical pathways. They are starting with *Synechocystis* as their model organism. The genome of this cyanobacterium was sequenced in Japan, representing the first genome sequence of a photosynthetic organism, and they are now working on a microarray project.

The progress made so far by Japanese members of the consortium working on the **functional genomics of *B. subtilis*** was described by Naotake Ogasawara (Nara Institute, Japan). 42% of *B. subtilis* genes have no known function and over half of these are unique to *B. subtilis*. The functional analysis project uses a combination of gene disruption (or regulated expression for essential genes), phenotypic analysis and gene expression profiling (using Northern blotting and lacZ reporter gene fusions). The Northern blot analysis is almost

Copyright © 2000 John Wiley & Sons, Ltd.

complete, and they have been able to detect messages for 80% of the genes tested. Several probes detected shorter messages than expected, hence the team suggest that there may be a previously unknown transcriptional termination signal. Almost all of the ~900 mutants have been produced and ~40% of the 800 tested so far show a discernable phenotype in their tests. The plan is to try to cluster genes based on their phenotypes under various stresses, including temperature, ethanol, SDS, salt and xylose stress.

Pierre Legrain (Hybrigenics, France) presented a **two-hybrid protein interaction assay strategy that uses prey libraries** to give high throughput and allow determination of binding domains. By using libraries expressing parts of prey genes to define binding domains of prey proteins, the libraries allow screening of up to 10 million preys in one experiment and tens of baits can be done in parallel, adding more throughput. Software tools assign a score for the reliability of each interaction and generate protein interaction maps (PIMs), these can be explored using a visualization tool, PIMrider, which can link to external databases to glean further information. The system has been applied to *Helicobacter pylori* and to date, over 1200 interactions linking more than 800 proteins make up the PIM of *H. pylori*. A small selection of these interactions have been tested for conservation in *E. coli*, with promising results.

The **yeast functional genomics** project was described by Steve Oliver (University of Manchester, UK). At the genomics level, many genes have been disrupted in the 'six-pack' node of EUROFAN I, this showed that ~14% of yeast genes were essential, but also that many genes had no discernible phenotype. Transcriptome analysis was initiated using Northern blots. This phase has been completed but, despite this, no message can be detected for 20% of genes in yeast grown under a variety of physiological conditions. The second phase of the transcriptome analysis uses 'macroarrays' to compare global transcription patterns under different conditions, such as carbon starvation and nitrogen starvation. Clustering of genes that are regulated in the same way have been used to find known transcription-factor-binding sites and to predict new ones. Finding a known site upstream of an ORF can be used to infer function. Proteomics studies include mass spectrometry of protein samples separated by either 2D gels or liquid chromatography and chemical derivatization steps to improve the speed and accuracy of protein identification. Metabolite samples from the same experiments are analysed by NMR Spectroscopy to provide 'fingerprints' that can be used to cluster mutants of novel genes with those of genes with known metabolic roles.

The Japanese **functional genomics project for *E. coli*** was presented by Hirotada Mori (Nara Institute, Japan). The project involves making deletion and disruption mutants, cloning genes for the production of a microarray, and proteomics and bioinformatics studies. Known genes are being cloned as C-terminal GFP fusions with N-terminal 6xHis tags. These allow the determination of the subcellular localization of the proteins and purification of the proteins. A new vector has been developed that allows insertion of *lacZ* and an arabinose-responsive promoter between a chosen ORF and its promoter. This allows use of β-galactosidase to monitor the activity of the promoter and regulation of the expression of the gene using arabinose media. Another part of the project involves using a phage disruption system to generate mutants for phenotypic analysis. Microarrays have been constructed and are being used to assess the effects of knocking out putative transcription factors.

### Session E — structural genomics

Sung-Hou Kim (University of California, USA) presented a case study of the **application of structural genomics to *Methanococcus jannaschii***. They chose this archaean because, as a thermophile, it was believed that it will have highly stable proteins that should be easy to purify and crystallize. The group determined the crystal structures of two hypothetical proteins and one with inferred function. The structure of the first protein had an ATP bound, despite the lack of a conventional ATP binding domain. The fold is similar but not the same as the previously known one, and only hydrolyses ATP in the presence of *Methanococcus* extract at 80°C, so it is probably a molecular switch. The second protein had a completely new fold but had weak similarities to many nucleotide-binding domains. Kim's group found that it binds to XTP and ITP and hydrolyses these incorrect bases. The third protein was classified as a small

Copyright © 2000 John Wiley & Sons, Ltd. 

heat shock protein and is found in archaea, prokaryotes and eukaryotes. Its crystal structure is identical to an immunoglobulin fold, despite the lack of sequence homology.

Alfonso Valencia presented a method for **detecting interacting protein pairs from sequence information**. This *in silico* two-hybrid (i2h) method is based on the theory that mutations in one partner of an interacting pair can select for mutations in the other partner. His group searched for these correlated mutations in multiple alignments of known interacting protein pairs to see if such an effect could be observed. Not only could these effects be seen, but they could be used to identify residues close to the interacting regions of the proteins. The group hope to extend this work to cover whole genomes, by combining prediction methods and by including more genomes in their alignments, as they are completed.

### Session F — evolution and comparative genomics

Russell Doolittle discussed the reasons for the controversy surrounding the phylogenetic relationship of the three principal domains of living organisms; Archaea, Bacteria (eubacteria) and Eukarya (eukaryotes) in a talk entitled **'searching for the common ancestor'**. The phylogenetic tree based on rDNA sequence data was proposed by Woese in 1987, which produced the three groupings mentioned previously. Since then, there has been great debate over which of the three possible trees is the right one (Figure 3A). It was thought that the whole genome sequencing initiative would yield the type of data required to find an answer. However, numerous horizontal transfer events have occurred which have served to confuse matters further. Data exist to support each pair being the most closely related; only archaea and eubacteria have circular chromosomes and some of their genes are organized into operons; eubacteria and eukaryotes make ester lipids, whereas archaea have ether lipids; only archaea and eukaryotes have VATPase, etc. Russell favours a model in which eukaryotes branched off first, and were later invaded by mitochondria and then chloroplasts, also gaining genes such as PFK and elongation factors by horizontal transfer, subsequent to the divergence of archaea and eubacteria (Figure 3B).

Siv Andersson (Uppsala University, Sweden) presented the implications of the study of **the *Rickettsia prowazekii* genome** for our understanding of the origin of the mitochondrion. *Rickettsia* is an α-proteobacterium; these appear to be the closest known relatives to mitochondria. As much as 20% of the genome is regarded as junk, with many pseudogenes and genes that appear to be undergoing degradation. These genes may once have been active; their GC content is the same as the rest of the genome. Only *Rickettsia* and *Chlamydia* have ATP/ADP translocases. However, although all mitochondrial ATP/ADP translocases are similar, they are not like the *Rickettsia* one. Andersson does not think it was this gene that provided the main selective advantage requiring recruitment of the protomitochondrion. Rather, she suggests that it could relate to the toxicity of oxygen. Since the bacteria take up oxygen, they might have associated with eukaryote cells, thereby reducing their considerable oxygen load. At some point, the bacteria was taken up into the cell and the transporter evolved later, since this would be beneficial to the eukaryote, allowing it to use ATP produced by the mitochondrion. Other genes were subsequently exchanged in both directions.

Steve Benner (University of Florida, USA) presented the new developments of the 'Darwin' software package, first published in 1992, which makes **functional inferences using reconstructed evolutionary biology** data. The database consists of the families of modules from all the currently known genomes ($10^4$–$10^5$) combined with data from other areas, including physiology and palaeontology, to produce models of the evolutionary history of each family. The database can be used to find distant homologues, since proteins performing analogous functions retain structural homology long after the sequence diverges. In highly related proteins, there is an increased frequency of amino acid changes caused by point mutations, those where the codon changes by just one base. The program takes into account non-stochastic behaviour such as this. Other effects, such as the variation in mutation rates of surface amino acids compared to internal ones, are also taken into account in their predictions, which they believe give better structural data than Chou-Fasman type algorithms. Although these predictions are not good enough to give 1.5Å structures,

Copyright © 2000 John Wiley & Sons, Ltd.

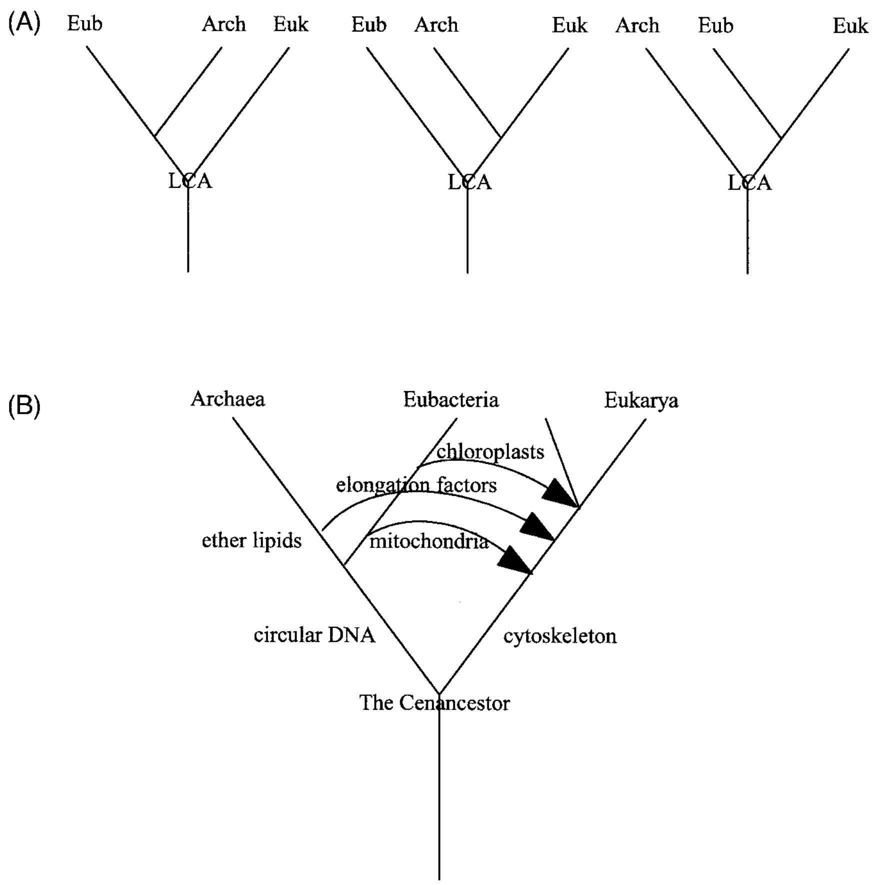

**Figure 3.** (A) The three possible trees indicating the relationship between archaea, eubacteria and eukaryotes. Eub, Eubacteria; Arch, Archaea; Euk, Eukary; LCA, last common ancestor. (B) A figure based upon the model proposed by Russell Doolittle in his presentation. The arrows represent occurrences of horizontal transfer and invasion by organisms which became organelles they do allow detection of distantly related structures.

Bernard Dujon (Institut Pasteur, France) reported on the results of a large-scale **comparative analysis of a variety of yeast species.** The group obtained 50 000 reads, giving 20–40% coverage of the yeasts chosen to represent a range of phylogenetic distances from *Saccharomyces cerevisiae*. The insert size of the clones used was chosen to maximize the chance of hitting two *S. cerevisiae* genes. Both ends of each insert were sequenced. Only 3500 *S. cerevisiae* ORFs have matches in the complete genomes released so far. Several of the 'unique' genes find a match in the *Schizosaccharomyces pombe* sequence, and when these data on the other yeasts are included, the number of putative ascomycete-specific genes rises to 1200. The group feel that 5600 is a more accurate gene count for *S. cerevisiae*, based on these results. The data were also used to search for synteny between *S. cerevisiae* and the other yeast genomes. As expected, the degree of synteny observed decreases with phylogenetic distance from *S. cerevisiae* (from 97% in *S. bayanus* to only 10% in *Yarrowia lipolytica*) and the number of inversions detected increases with phylogenetic distance from *S. cerevisiae* (0–5% inversions between syntenic pairs in all strains up to *Kluyveromyces marxianus*, to 30% inversions for the most distantly related strains). From these data, they predict that a minimum of 6000 rearrangements must have happened since the divergence between *Y. lipolytica* and *S. cerevisiae*, this number decreases with decreasing phylogenetic distance from *S. cerevisiae*.

Copyright © 2000 John Wiley & Sons, Ltd. 

**Comparative *Mycobacterium* genomics** was the topic of Stewart Cole's (Institut Pasteur, France) presentation. The comparison in question was between *Mycobacterium tuberculosis*, whose genome sequence was completed two years ago and *M. leprae* (the causative agent of leprosy), whose genome is almost completed. *M. leprae* has the smallest mycobacterial genome at 3.3 Mb and also has the lowest GC content (57.8%) and a very low gene density. There are 1501 *M. leprae* genes in common with *M. tuberculosis*, ~100 *M. leprae*-specific genes and ~800 pseudogenes. Interestingly, ~1700 *M. tuberculosis* genes appear to be absent from *M. leprae*. The comparison of the two genomes gives 65 segments of conserved gene order. This large number of rearrangements is thought to be due to recombinations between repeated sequence elements. Compared to *M. tuberculosis*, *M. leprae* appears to have lost catabolic functions; there are no P450 genes, the NADH oxidase operon is almost completely deleted, it has no siderophores, and it is recombination-deficient. So, *M. leprae* has a scrambled genome, which shows evidence of downsizing and decay, compared to *M. tuberculosis*, and could represent a naturally defined core mycobacterial gene set.

---

The Meeting Highlights of *Comparative and Functional Genomics* aim to present a commentary on the topical issues in genomics studies presented at a conference. The Meeting Highlights are invited, and each represents a personal critical analysis of the current reports, which aims at providing implications for future genomics studies.

*The*
**Genomics**
*Website at Wiley*

**www.wiley.co.uk/genomics**

The **Genomics** website at Wiley is a new and **DYNAMIC** resource for the genomics community, offering **FREE** special feature articles and new information **EACH MONTH**.

Find out more about *Comparative and Functional Genomics*, and how to view all articles published this year **FREE OF CHARGE**!

Visit the **Library** for hot books in Genomics, Bioinformatics, Molecular Genetics and more.

Click on **Primary Research** for information on all our up-to-the minute journals, including: *Genesis*, *Bioessays*, *Gene Function and Disease*, and the *Journal of Gene Medicine*.

Let the **Genomics** website at Wiley be your **guide** to genomics-related web sites, manufacturers and suppliers, and a calendar of conferences.

WILEY

1516

Copyright © 2000 John Wiley & Sons, Ltd.

